# Determining optimal planning target volume and image guidance policy for post-prostatectomy intensity modulated radiotherapy

**DOI:** 10.1186/s13014-015-0467-8

**Published:** 2015-07-26

**Authors:** Linda J. Bell, Jennifer Cox, Thomas Eade, Marianne Rinks, Alan Herschtal, Andrew Kneebone

**Affiliations:** Radiation Oncology Department, Northern Sydney Cancer Centre, Royal North Shore Hospital, St Leonards, NSW Australia; Faculty of Health Sciences, University of Sydney, Lidcombe, NSW Australia; Northern Clinical School, University of Sydney, St Leonards, NSW Australia; Department of Biostatistics and Clinical Trials, Peter MacCallum Cancer Centre, Melbourne, VIC Australia; Present address: Radiation Oncology, Shoalhaven Cancer Care Centre, Illawarra Shoalhaven Local Health District, Nowra, NSW Australia

**Keywords:** Post-prostatectomy radiotherapy, PTV, IGRT, CBCT, Geographic miss, Soft tissue matching

## Abstract

**Background:**

There is limited information available on the optimal Planning Target Volume (PTV) expansions and image guidance for post-prostatectomy intensity modulated radiotherapy (PP-IMRT). As the prostate bed does not move in a uniform manner, there is a rationale for anisotropic PTV margins with matching to soft tissue. The aim of this study is to find the combination of PTV expansion and image guidance policy for PP-IMRT that provides the best balance of target coverage whilst minimising dose to the organs at risk.

**Methods:**

The Cone Beam CT (CBCT) images (*n* = 377) of 40 patients who received PP-IMRT with daily online alignment to bony anatomy (BA) were reviewed. Six different PTV expansions were assessed: 3 published PTV expansions (0.5 cm uniform, 1 cm uniform, and 1 + 0.5 cm posterior) and 3 further anisotropic PTV expansions (Northern Sydney Cancer Centre (NSCC), van Herk, and smaller anisotropic). Each was assessed for size, bladder and rectum coverage and geographic miss. Each CBCT was rematched using a superior soft tissue (SST) and averaged soft tissue (AST) match. Potential geographic miss was assessed using all PTV expansions except the van Herk margin.

**Results:**

The 0.5 cm uniform expansion yielded the smallest PTV (median volume = 222.3 cc) and the 1 cm uniform expansion yielded the largest (361.7 cc). The Van Herk expansion includes the largest amount of bladder (28.0 %) and rectum (36.0 %) and the 0.5 cm uniform expansion the smallest (17.1 % bladder; 10.2 % rectum). The van Herk PTV expansion had the least geographic miss with BA matching (4.2 %) and the 0.5 cm uniform margin (28.4 %) the greatest. BA matching resulted in the highest geographic miss rate for all PTVs, followed by SST matching and AST matching. Changing from BA to an AST match decreases potential geographic miss by half to two thirds, depending on the PTV expansion, to <10 % for all PTV expansions. When using the smaller anisotropic PTV expansion, AST matching would reduce the geographic miss rate from 21.0 % with BA matching down to 5.6 %.

**Conclusions:**

Our results suggest the optimal PTV expansion and image guidance policy for PP-IMRT is daily average soft tissue matching using CBCT scans with a small anisotropic PTV expansion of 0.5 cm in all directions apart from a 1 cm expansion in the anterior-posterior direction in the upper prostate bed. Care must be taken to ensure adequate training of Radiation Therapists to perform soft tissue matching with CBCT scans.

## Introduction

Standard image verification practice in PP-IMRT is daily alignment to bony anatomy, although the independent movement of the prostate bed makes bony anatomy a poor surrogate for prostate bed motion [1, 2]. The upper prostate bed moves more than the lower prostate bed, making it difficult to correct equally in both regions, due to the tilt and deformation generated by variation in bladder and rectum volumes [3–5]. Currently the European Organisation for Research and Treatment of Cancer (EORTC) Radiation Oncology Group recommends a minimum of weekly portal imaging matched to bony anatomy with correction protocols [6]. Matching to the prostate bed rather than to bony anatomy might enable smaller PTV expansions to be used, helping reduce dose to surrounding critical structures.

Consensus guidelines assist Radiation Oncologists to define the clinical target volume (CTV) for post-prostatectomy intensity modulated radiotherapy (PP-IMRT) [6–8]. There is, however, little evidence to inform planning target volume (PTV) expansions in this setting. The EORTC Radiation Oncology Group recommends that a minimum of a 0.5 cm uniform PTV expansion be used [6], while The Australian and New Zealand Radiation Oncology Genito-Urinary Group (FROGG) have recommended a uniform 1 cm margin plus an acceptable reduction to a 0.5 cm posterior expansion, especially if the rectal dose exceeds guidelines [7].

The aims of this study were to (1) determine the best PTV expansion for post-prostatectomy intensity modulated radiotherapy, taking into consideration PTV volume, the percent of critical structures being treated, and bony anatomy geographic miss rates, (2) determine the ideal image guidance policy for post-prostatectomy intensity modulated radiotherapy, and (3) determine if soft tissue matching can enable smaller PTV expansions to be used.

## Methods

Ethics approval was granted from the Northern Sydney Central Coast Health Human Research Ethics Committee (1103-082M) and the University of Sydney Human Research Ethics Committee (13721) before the commencement of this study.

A retrospective study was conducted where the images of 40 patients who had received PP-IMRT at the Northern Sydney Cancer Centre (NSCC) with an image guided-intensity modulated radiotherapy technique were sequentially selected over the period 2009 to 2011. Patients had surgical clips in the upper and lower portions of the treatment volume and had at least one CBCT taken during treatment.

Prior to treatment simulation, patients were placed on a low residue diet with magnesium to try to maintain an empty rectum and were instructed to have a comfortably full bladder and an empty rectum by emptying their bladder and bowels and then drinking 600 ml of water 1 h prior to simulation. A BladderScan® (Verathon Incorporated, Bothell, WA, USA) ultrasound device was used to check that the patient had adequate bladder filling prior to the planning CT. If the rectal diameter in the anterior-posterior direction was greater than 3.5 cm on the planning CT the patient was given an enema and a new planning scan was acquired. During treatment all patients followed the same bladder & rectal protocol with feedback from regular CBCT scans.

Radiation Oncologists contoured the CTV on each of the planning CT scans according to the FROGG consensus guidelines [7]. The CTV is split into two sections, the upper and lower prostate bed, divided at the level where the CTV moves away from the pubic symphysis. This is to allow for anisotropic PTV expansions to be applied easily in the treatment planning system.

CBCT scans were taken using the on-board imager® (Varian Medical Systems, Palo Alto, CA, USA) on the first 3 fractions and then weekly for the remainder of the treatment course, according to standard departmental protocol. The CBCT scans taken in the first week of treatment and one CBCT scan from each subsequent week were analysed. If more than one CBCT was taken per fraction due to an intervention, only the first CBCT acquired was analysed. When patients were replanned during the treatment course to correct patient changes or other systematic issues, the CBCT scans after the replan commenced treatment were matched to the replan planning CT scan.

Six different PTV expansions were delineated on the planning CT scans using the Eclipse treatment planning system (Varian Medical Systems, Palo Alto, CA, USA). These were three PTV expansions from the European and Australian post-prostatectomy published guidelines [6, 7] and three further anisotropic PTV expansions. The details of the expansions are shown in Table 1. The first was an expansion developed by continued review of CBCT since 2007 at the NSCC and in clinical practice in this unit since 2010. Secondly, an expansion was calculated using the van Herk margin recipe [9] from previously collected movement data [3], and the third expansion applied was a smaller alternative anisotropic PTV margin, based on the theory that soft tissue matching might allow greater reduction of margins.

**Table 1:** Planning target volume expansions

Area of prostate bed	Direction of expansion	PTV Name					
		0.5 cm uniform (cm)	1 cm uniform (cm)	1 + 0.5 cm posterior (cm)	NSCC (cm)	van Herk (cm)	Smaller Anisotropic (cm)
Upper	Anterior	0.5	1	1	1.5	1.9	1.0
	Posterior	0.5	1	0.5	1	1.9	1.0
	Superior	0.5	1	1	1	1.1	0.5
	Inferior	0.5	1	1	0.5	1.1	0.5
	Left	0.5	1	1	1	0.5	0.5
	Right	0.5	1	1	1	0.5	0.5
Lower	Anterior	0.5	1	1	0.8	0.7	0.5
	Posterior	0.5	1	0.5	0.8	0.7	0.5
	Superior	0.5	1	1	0.8	0.7	0.5
	Inferior	0.5	1	1	0.8	0.7	0.5
	Left	0.5	1	1	0.8	0.4	0.5
	Right	0.5	1	1	0.8	0.4	0.5

Details of the planning target volume expansions used

*Abbreviations*: *cm* centimetre; *NSCC* Northern Sydney Cancer Centre

The volume irradiated (cc) and the volumes of bladder and rectum included for each PTV expansion were calculated by measuring the volume of each PTV structure and the overlapping volume of bladder and rectum in Eclipse (Varian Medical Systems, Palo Alto, CA, USA).

The CBCT scans and planning CT scan were matched to bony anatomy (BA) using Offline Review (Varian Medical Systems, Palo Alto, CA, USA). Prostate bed coverage by each PTV was reviewed for geographic miss, defined as the location of any soft tissue, surgical clip, posterior bladder wall, or anterior rectal wall, classified as CTV, that was outside the PTV. In addition to BA, two other soft tissue matching techniques were tested: (1) rematching the CBCT scans and planning CT scan to the soft tissue in the superior prostate bed on the first slice where the CTV moves away from the posterior pubic symphysis, which would be a simple and fast method of matching on treatment, called a superior soft tissue (SST) match and (2) rematching the scans using an averaged soft tissue (AST) match to account for movement of the prostate bed on all CT slices. Geographic miss was assessed for all PTV margins.

The bladder and rectum volumes and the rate of geographic miss for each matching technique were used to determine the optimal PTV expansion and image guidance policy. Microsoft Excel® (Microsoft Corporation, Redmond, WA, USA) and Statistical Package for the Social Sciences® (SPSS) (IBM Corporation, Armonk, NY, USA) were used to conduct statistical analyses on the data collected.

## Results

A total of 377 CBCT scans were reviewed in 40 post-prostatectomy patients (median number of CBCTs per patient was 9, range of 8 to 11). Forty-five planning CT scans were reviewed.

### PTV size

The 0.5 cm uniform expansion had the smallest PTV volume (median = 222.3 cc), followed by smaller anisotropic (238.9 cc), NSCC (331.5 cc), 1 + 0.5 cm posterior (337.7 cc), van Herk (354.1 cc), and 1 cm uniform (361.7 cc).

### Volume of bladder and rectum inside the PTV

**Fig. 1:**
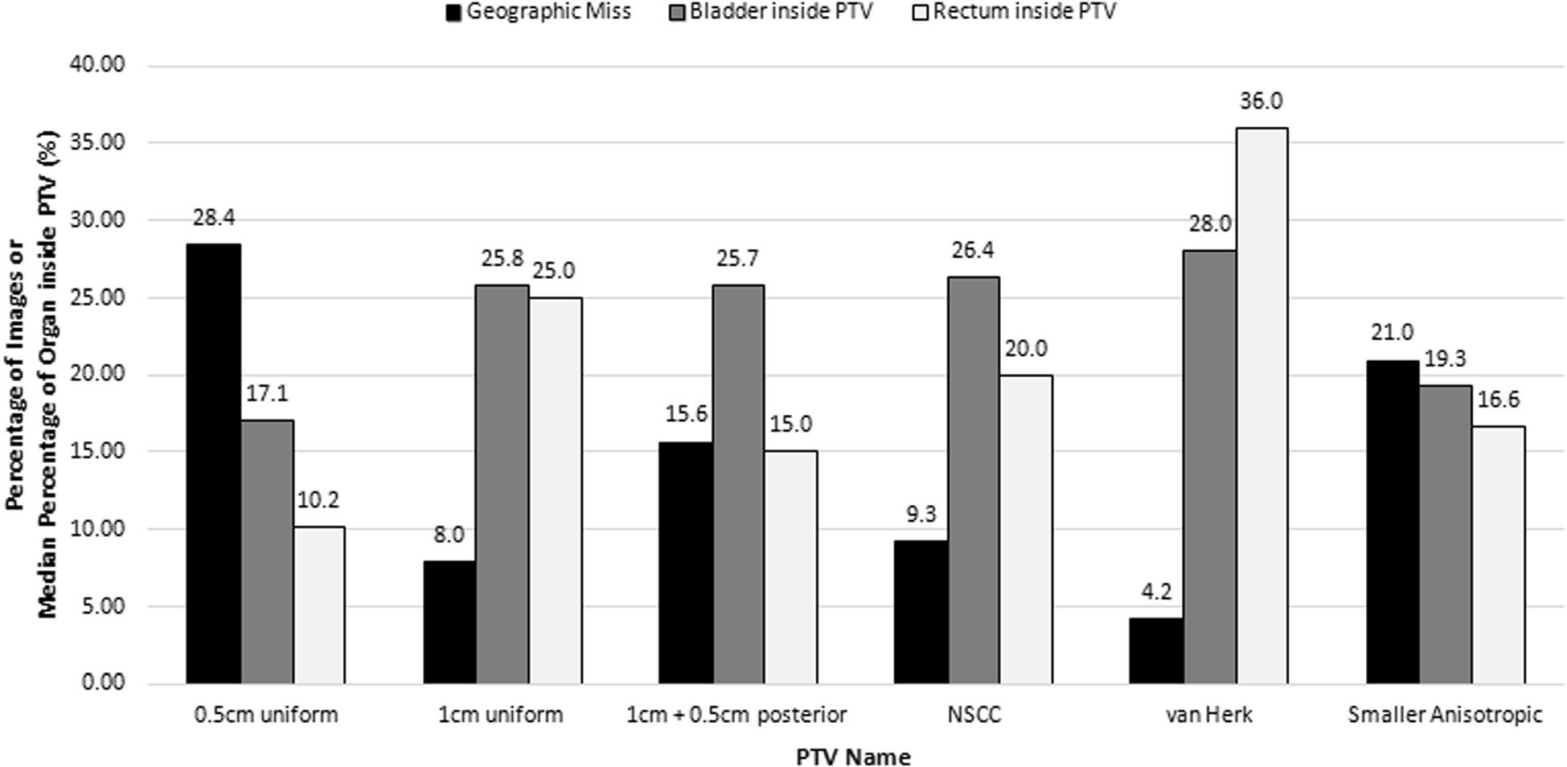
Rates of geographic miss with volume of bladder and rectum inside the PTV using bony anatomy matching. The percentage of all bony anatomy matched images that showed a geographic miss for each PTV compared to the percentage of the bladder and rectum located inside the PTV

The volume of bladder and rectum inside each PTV is shown in Fig. 1. The NSCC PTV had only 1 % more bladder in the PTV than the 1 cm uniform expansions despite having a 0.5 cm larger PTV expansion anteriorly. The larger expansions of the van Herk PTV covered much of the rectum. The NSCC PTV had 5 % less rectum in the volume than the 1 cm uniform PTV although the upper prostate bed margin is the same, due to the decreased posterior expansion in the lower prostate bed.

### Rate of geographic miss using different matching techniques and PTVs

**Fig. 2:**
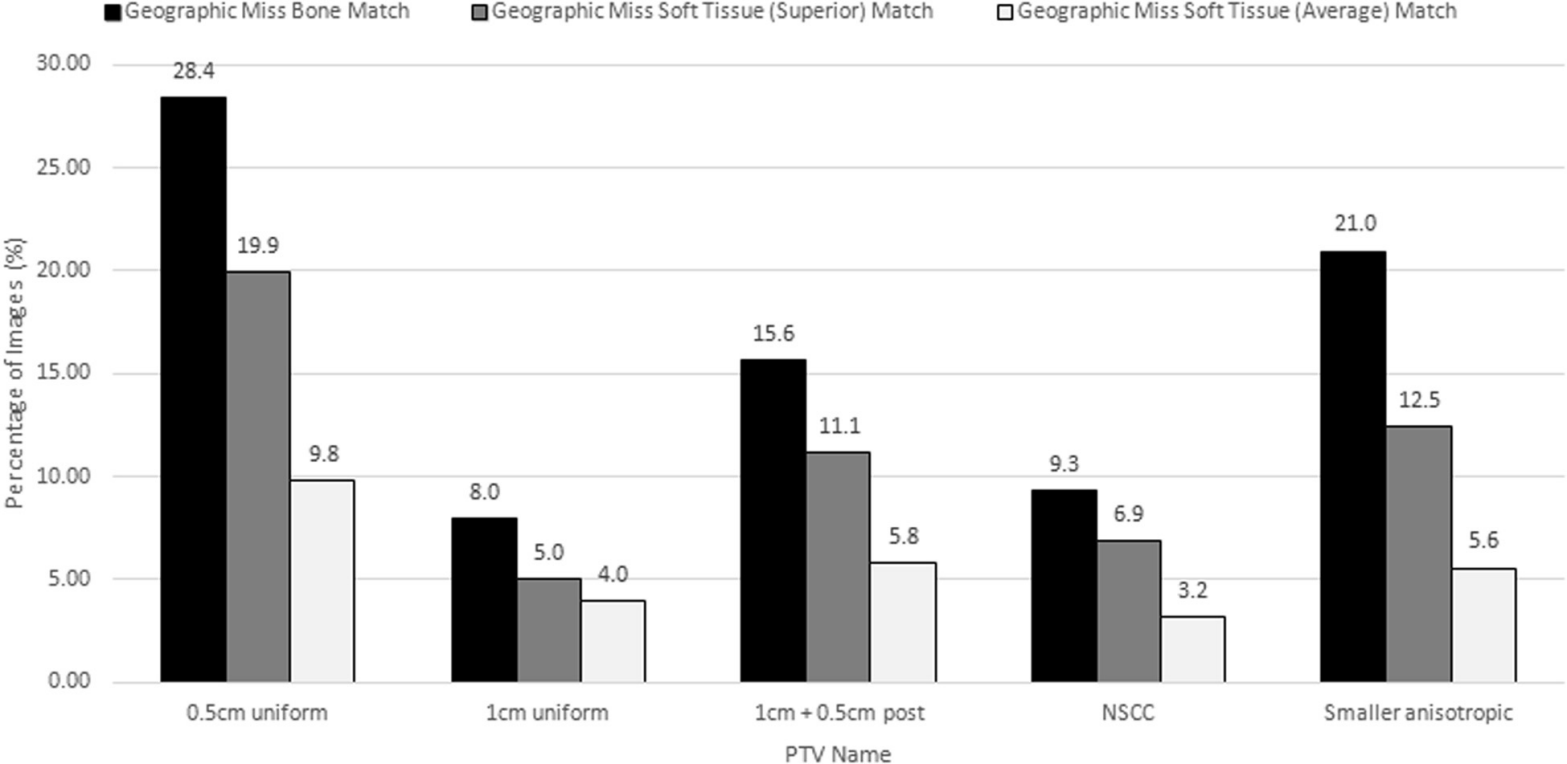
Geographic miss rates using different matching techniques and PTV expansions. The percentage of images displaying a geographic miss for three matching techniques (bone, superior soft tissue, and average soft tissue) for different PTV expansions

The rate of geographic miss using three different matching techniques and five different PTV expansions (as previously described, excluding the van Herk margin) is shown in Fig. 2. The van Herk margin was excluded because this PTV covered a prohibitive amount of the rectum, so could not be implemented clinically. With all matching types, rates of geographic miss increased as the PTV expansions became smaller.

BA matching resulted in the highest geographic miss rate for all PTVs, followed by SST matching then AST matching (see Fig. 2). Changing from a BA to an AST match decreased potential geographic miss by half to two thirds, depending on the PTV expansion. Changing soft tissue matching from the SST to the AST technique resulted in an approximate 50 % decrease in geographic miss for all PTVs except the 1 cm uniform expansion. The AST matching technique reduced geographic miss rates to below 10 % for all PTV expansions tested and to less than 6 % for four of the PTV expansions. Using the anisotropic NSCC PTV expansion with the AST matching technique would result in a 3.2 % geographic miss rate compared to 9.3 % with BA matching. The smaller anisotropic PTV expansions resulted in a slightly higher rate of geographic miss of 5.6 % (a 2.4 % increase) than the NSCC PTV expansion when using the AST match.

### Location of soft tissue geographic misses using BA or AST matching

**Fig. 3:**
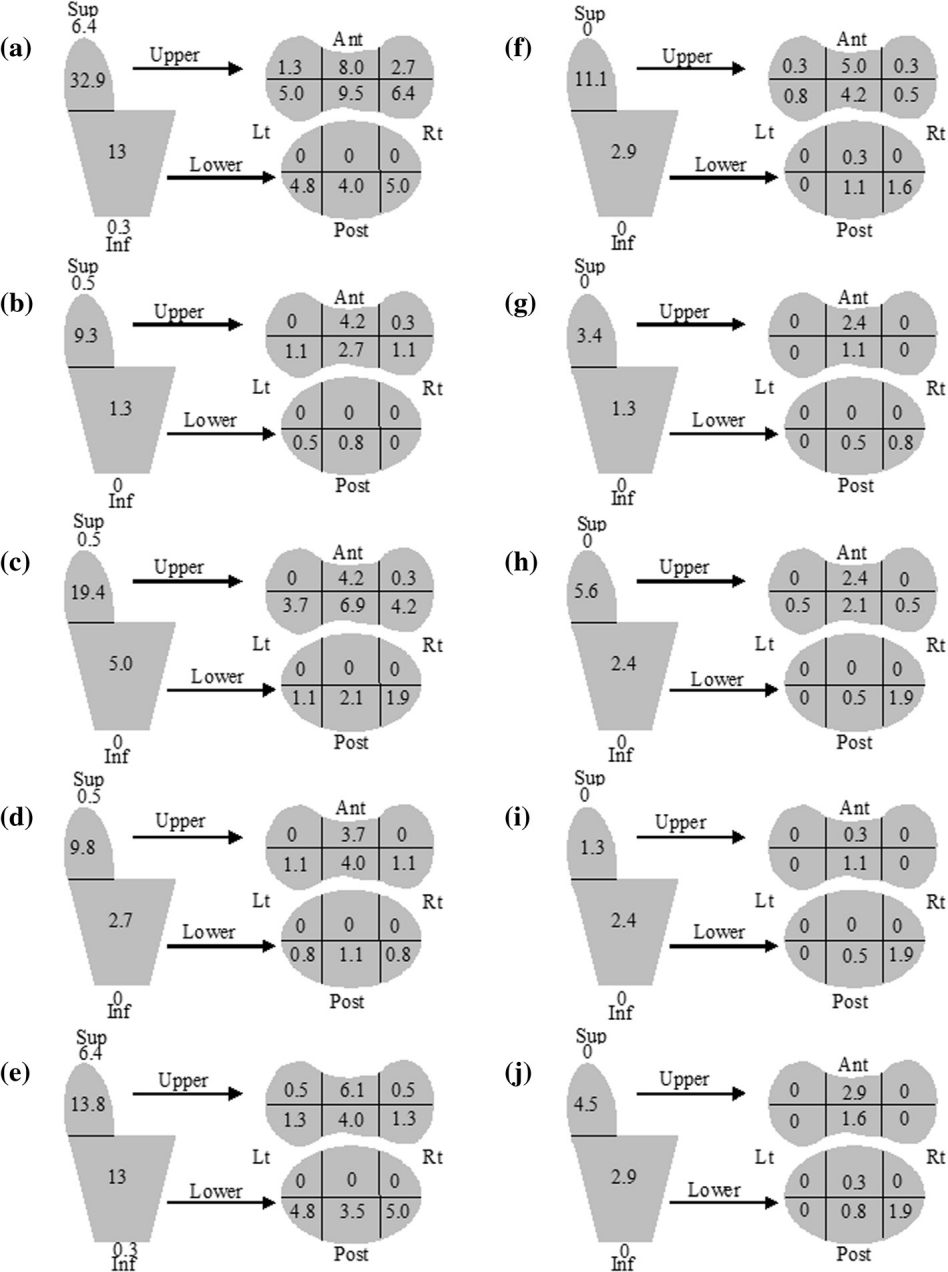
Area of prostate bed where soft tissue geographic miss occurred. Schematic diagrams of the areas of prostate bed where soft tissue geographic miss occurred for the 0.5 cm uniform (**a** & **f**), 1 cm uniform (**b** & **g**), 1 + 0.5 cm posterior (**c** & **h**), NSCC (**d** & **i**), and smaller anisotropic (**e** & **j**) PTV expansions when bony anatomy matching (*left column*) or averaged soft tissue matching (*right column*) was used. Numbers refer to total percentage of misses in each area over 377 images

The AST match reduced most lateral geographic misses compared with BA matching for all PTV expansions (see Fig. 3), with the majority of geographic misses, although reduced in number, in the central area of the PTV, especially in the upper prostate bed. AST matching resulted in a smaller increase in anterior geographic misses in the lower prostate bed than with BA matching, but the overall number of geographic misses was reduced.

### Combined evaluation of the PTV expansions and image guidance matching techniques

When evaluating PTV expansions, both the rates of geographic miss and the volume of critical structures treated were considered (Figs. 1 and 2). The van Herk PTV expansion had the smallest rate of geographic miss using BA matching (4.2 %). However it included the largest amount of bladder (28.0 %) and rectum (36.0 %), which would make it difficult to implement clinically, so it was eliminated from the study prior to assessing soft tissue matching. The 0.5 cm uniform PTV expansion treated the least bladder (17.1 %) and rectum (10.2 %) but had the highest geographic miss rate (BA = 28.4 %, SST = 19.9 %, AST = 9.8 %). The 1 cm uniform PTV expansion treated more rectum (25.0 %) than the NSCC PTV (20.0 %), but only decreased geographic miss by 1.3 % compared to the NSCC PTV. The 1 + 0.5 cm posterior PTV expansion covered a similar amount of bladder (25.7 %) to the NSCC and 1 cm uniform PTV expansions with less rectum (15.0 %), but it had nearly double the rate of geographic miss of the 1 cm uniform and NSCC PTV. The NSCC PTV also had a smaller median volume (331.5 cc) than the 1 cm uniform (361.7 cc) and 1 + 0.5 cm posterior (337.7 cc) expansions. With a geographic miss rate of 9.3 % with BA matching, this PTV expansion might be optimal, but geographic miss rate improved to 3.2 % with AST matching. The smaller anisotropic PTV expansion was similar to the 0.5 cm uniform PTV expansion for BA matching geographic miss and bladder and rectal volumes, but the geographic miss rate decreased to 5.6 % with AST matching. This expansion has the benefits of decreasing normal tissue and critical structure dose compared with the NSCC volume, with only a small increase in geographic miss.

### Discussion

Intensity modulated radiotherapy (IMRT) treatment techniques are desirable in the post-prostatectomy setting due to the ability to produce highly conformal plans with rapid dose fall off [10], enabling decreased dose to be delivered to surrounding critical structures such as the bladder and rectum. They are, however, susceptible to movement and geographic miss. Current published PTV guidelines assume uniform prostate bed movement, but recent findings of non-uniform prostate bed movement [3] support an argument for anisotropic PTV margins. The good soft tissue definition of CBCT imaging [11] could enable a change in image verification processes to a daily soft tissue matching approach which would further increase treatment accuracy and enable geographic misses to be identified and corrected before treatment.

Our study supports the theory that anisotropic expansions are the optimal PTV expansion in the PP-IMRT setting when BA, SST, or AST matched interfraction imaging is used. The size of the anisotropic PTV expansion will depend on the imaging matching technique applied.

If BA matching is used, the NSCC PTV expansion would be the best to use. It was the third smallest in volume (median = 331.5 cc), but treated a similar amount of bladder to the 1 cm uniform and 1 + 0.5 cm posterior PTV expansions, with the fourth smallest amount of rectum overall, and a BA match geographic miss rate of 9.3 % of images. With BA matching, the 1 cm uniform PTV covers more rectum but only decreases geographic miss by 1.3 %, and the 1 + 0.5 cm posterior PTV covers less rectum but nearly doubles the geographic miss rate.

When AST matching is used, the NSCC PTV expansion produces the lowest geographic miss rate of 3.2 %. Moreover, application of the AST technique could lead to a reduction in PTV expansions, such as the smaller anisotropic PTV expansion, which resulted in a 2.4 % increase in potential geographic miss of 5.6 % compared to 3.2 % with the NSCC PTV expansion. Using the smaller anisotropic margin also has the added benefit of reduced critical structure volumes inside the PTV compared to the NSCC PTV, with bladder reducing from 26.4 % to 19.3 % and rectum reducing from 20.0 % to 16.6 %. All matching techniques enable detection of geographic miss before treatment delivery if CBCT scans are taken. The averaged nature of the AST match also gives more flexibility to facilitate inclusion of all the soft tissue inside the PTV for treatment. The decreased dose to the surrounding critical structures achieved by the use of the smaller anisotropic PTV expansions could also enable dose escalation. Additionally, there is potential for a more adaptive radiotherapy approach with tailored margins for specific patients, although this would require further investigations into the predictive nature of geographic miss.

The bladder and rectum volumes inside each PTV were used to assess if planning critical structure limitations could be met. The planning protocol used in our department is: PTV (D95% = 100 % of Target Dose), rectum (V6500cGy < 17 %, V6000cGy < 20 %, V4000cGy < 40 %), and bladder (V6500cGy < 25 %, V4000cGy < 50 %). The NSCC PTV expansion is used clinically in our department and these planning objectives can be met in the majority of cases. Although no dosimetric analysis was carried out, it is clear that the volumes of rectum and bladder inside the 1 cm uniform and van Herk PTV expansions would make it difficult to meet these dose constraints.

Two-thirds of local recurrences after surgery occur at the anastomosis (of these 60 % were posterior, 20 % anterior, and 15 % lateral), 17 % in the retrovesical space, 10 % in the bladder neck and 10 % elsewhere [6]. Our data shows that, for all matching techniques, the most common areas of potential geographic miss coincide with these areas of highest risk of recurrence, i.e. posteriorly in the lower prostate bed and in the central upper prostate bed. Soft tissue matching does not change the area where geographic miss occurs, but it does result in reduced potential misses. It is also possible that the soft tissue matching process would result in the Radiation Therapists noticing a geographic miss prior to treatment delivery so intervention such as bladder or rectum emptying could occur. Daily CBCT soft tissue matching and/or the addition of implanted markers [12] could therefore reduce the occurrence of geographic miss, allowing smaller PTV volumes to be used.

It should be noted that these patients followed a bladder and rectal filling protocol. Patients were simulated with a comfortably full bladder and empty rectum, with an enema administered if the rectal diameter was greater than 3.5 cm. Enemas were not given during treatment but the same bladder and rectal preparation was used. If the PTV expansions and IGRT protocols recommended here were to be adopted the same bladder and rectal preparation would be required.

This study had some limitations. Although 377 CBCT images were reviewed, they only belong to 40 patients. Reviewing a larger number of patients could produce more robust results and give more information on the factors predictive of geographic miss.

The time it would take to acquire daily CBCT scans and to complete the soft tissue matching has not been assessed here. CBCT acquisition takes longer than standard orthogonal imaging acquisition and there could be a difference in the time it takes to complete the different matching techniques. This might increase the time required to treat each patient, at least during the initial implementation phase. Radiation Therapist training would also be necessary, but they have been shown to be adept after using a locally produced image atlas [13, 14].

CBCT imaging delivers more radiation dose to the patient than daily kV/kV imaging. It is estimated that a pelvic mode CBCT scan delivers 17.7 mGy compared with 3.54 mGy for kV/kV imaging [15]. This increased imaging dose needs to be considered before deciding to implement a daily CBCT imaging policy, but the higher accuracy within the context of the treatment dose probably outweighs this disadvantage. Departments also should ensure that the image quality of their CBCT scans is adequate for soft tissue matching. Structure identification on CBCT scans was assessed at NSCC and it was found that with training, the anterior rectal wall, posterior bladder wall and prostate bed surgical changes were well recognised on the majority of scans [14].

PTV expansion reduction should be done with caution and all errors that the PTV accounts for should be assessed before doing so. We used CBCT scans to assess motion, which only accounts for interfraction motion, and the effect of intrafraction motion has not been considered, although this is likely to occur during PP-IMRT. Klayton et al. [12] conducted an intrafraction motion study using radio-frequency transponders and found that bladder and rectum variability caused deformation of the prostate bed. The 5 mm tracking limit was exceeded for at least 30 s in 11 % of all fractions, and 15 % of all treatments were interrupted for repositioning. Intrafraction motion should therefore be considered before reducing PTV expansions.

**Fig. 4:**
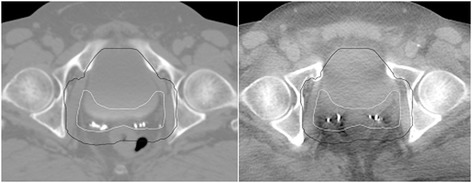
Increased rectum inside PTV. Geographic miss could be avoided using soft tissue matching in a number of instances. A planning CT (*left*) and CBCT scan (*right*) are shown in this example. In this case averaged soft tissue matching would have allowed a geographic miss to be corrected using the 1 cm uniform PTV expansion (*black line*), but a large amount of rectum would have been treated. The white line is the CTV. Critical structure avoidance therefore needs to be reviewed when matching online

Another limitation of this study is that only geographic miss was assessed and not the encroachment of the PTV coverage onto surrounding critical structures. Geographic miss could be avoided in some CBCT scans but it resulted in an unacceptable amount of the rectum potentially being treated (see Fig. 4). Training of the Radiation Therapists is essential for online soft tissue matching. When completing an online soft tissue match, Radiation Therapists should assess both the coverage of the prostate bed and whether the coverage of the critical structures is too great. If the critical structures are included too much in the PTV, intervention can occur before treatment is delivered, such as filling or emptying the bladder, emptying the rectum, or rescanning and replanning.

## Conclusions

This study has shown daily alignment to BA might underdose important areas at risk of recurrence. Larger PTV margins can reduce this risk, but large volumes of bladder and rectum will then be in the PTV, making planning difficult. Anisotropic margins such as the NSCC expansion should be considered in the post-prostatectomy setting when daily bony alignment is used. The optimal image guidance policy for post-prostatectomy intensity modulated radiotherapy is daily AST matching using CBCT scans, which works best with an anisotropic PTV expansion. Daily soft tissue matching has the potential to allow reduced PTV expansions and enable an adaptive radiotherapy approach to be implemented. Care must be taken to ensure adequate training of radiation therapists to perform online soft tissue matching with CBCT scans.

## Abbreviations

PTV: Planning target volume
PP-IMRT: Post-prostatectomy intensity modulated radiotherapy
CBCT: Cone Beam CT
BA: Bony anatomy
NSCC: Northern Sydney Cancer Centre
SST: Superior soft tissue
AST: Averaged soft tissue
EORTC: European Organisation for Research and Treatment of Cancer
CTV: Clinical target volume
FROGG: Australian and New Zealand Radiation Oncology Genito-Urinary Group
IMRT: Intensity modulated radiotherapy
